# Copy number variations in high and low fertility breeding boars

**DOI:** 10.1186/s12864-015-1473-9

**Published:** 2015-04-10

**Authors:** Tamas Revay, Anh T Quach, Laurence Maignel, Brian Sullivan, W Allan King

**Affiliations:** University of Guelph, Ontario Veterinary College, Department of Biomedical Sciences, 50 Stone Rd E, Guelph, ON N1G 2W1 Canada; Canadian Centre for Swine Improvement Inc. (CCSI), Central Experimental Farm, Building #75, 960 Carling Avenue, Ottawa, ON K1A 0C6 Canada

**Keywords:** Pig, Boar, Low fertility, High fertility, Hypoprolific, Hyperprolific, Copy number variation, CNV, SNP50K, Markers of fertility

## Abstract

**Background:**

In this study we applied the extreme groups/selective genotyping approach for identifying copy number variations in high and low fertility breeding boars. The fertility indicator was the calculated Direct Boar Effect on litter size (DBE) that was obtained as a by-product of the national genetic evaluation for litter size (BLUP). The two groups of animals had DBE values at the upper (high fertility) and lower (low fertility) end of the distribution from a population of more than 38,000 boars. Animals from these two diverse phenotypes were genotyped with the Porcine SNP60K chip and compared by several approaches in order to prove the feasibility of our CNV analysis and to identify putative markers of fertility.

**Results:**

We have identified 35 CNVRs covering 36.5 Mb or ~1.3% of the porcine genome. Among these 35 CNVRs, 14 were specific to the high fertility group, while 19 CNVRs were specific to the low fertility group which overlap with 137 QTLs of various reproductive traits. The identified 35 CNVRs encompassed 50 genes, among them 40 were specific to the low fertility group, seven to the high fertility group, while three were found in regions that were present in both groups but with opposite gain/loss status. A functional analysis of several databases revealed that the genes found in CNVRs from the low fertility group have been significantly enriched in members of the innate immune system, Toll-like receptor and RIG-I-like receptor signaling and fatty acid oxidation pathways.

**Conclusions:**

We have demonstrated that our analysis pipeline could identify putative CNV markers of fertility, especially in case of low fertility boars.

**Electronic supplementary material:**

The online version of this article (doi:10.1186/s12864-015-1473-9) contains supplementary material, which is available to authorized users.

## Background

Pork is the most consumed meat in the world [1], thus high prolificacy of breeding animals represent a very important economic factor for the industry. As pigs are polytocous species, litter size is a direct measure of efficient fertilization and successful breeding. As a consequence, various litter size related traits are incorporated into genetic improvement programs with high economic importance. Genetic variability in genes with predicted reproductive functions and genotypes of linked SNP markers have been explored to identify hundreds of QTLs [2] and these markers have been successfully used to increase the rate of genetic gains. It is also known that large structural variations, such as chromosome rearrangements are major etiologic factors behind reproductive dysfunction and eradication of carriers could help in efficient and economical breeding [3]. Smaller sized genome rearrangements, such as deletions or duplications that disrupt the balance in genome integrity and result in copy number variations (CNVs) represent a novel type of molecular marker [4]. This class of structural variations have become the focus of research since its discovery [5,6] and in particular the recognition that a surprisingly high proportion of the human genome is involved in CNVs, potentially affecting gene expression and phenotype [7]. Since then, numerous studies described CNV in human populations and the current Database of Genomic Variants contains ~110,000 CNVs, covering 71.5% of the human genome. Also, the 90% of transcripts and 79% of microRNA loci are overlapped by CNVs [8]. Association of CNVs to disease states have also been attempted leading to the identification of putative markers involved in the development of various cancers, neurological disorders, recessive diseases, etc. [9,10].

Recently, this level of genome variability has also been investigated in domestic animals including cattle, pig, goat, sheep, horse, dog and chicken [11] and hold promise to become useful markers for genetic selection [4,12]. The first insight into CNV content of the porcine genome was from a study that involved only four chromosomes due to the difficulty of array CGH (aCGH) platform design [13]. Recently, with the availability of a more refined genome assembly, genome-wide high density oligonucleotide CGH arrays could be designed and used to investigate pigs from many different breeds[14,15], but it also opened the possibility of investigating CNVs from individual whole genome sequences [16,17].

The applicability of SNP genotyping arrays for the estimation of DNA copy numbers have made the Porcine SNP60k chip [18] the method of choice for several other research projects. Ramayo-Caldas et al. [19] have identified 49 CNVRs in 55 pigs, while 565 CNVRs have been described in a study of nearly 1700 pigs from 18 populations [20]. Wang et al. [21] investigated a large population of Large White x Minzhu pigs and described 249 CNVRs, while Fernandez et al. [22] investigated a highly inbred Iberian strain and found 65 CNVRs. Based on the studies using the SNP60k chip, CNVs cover 16.08% of the porcine genome [22]. This is a fraction of CNVR length reported in humans, most probably due to the smaller number of animals investigated and the less refined genome assembly and screening tools available, leaving much to discover.

Most of the available porcine CNV studies contain functional annotations of the gene content of identified CNVRs and provide important descriptions of new individual or breed specific variants with slightly different estimates of this level of genome variation in pig populations. Furthermore, Chen et al. [20] has associated several meat and carcass quality traits (QTL) with CNVRs and identified seven candidate genes potentially affecting these traits. Also, six CNVRs contained significant SNPs for several meat quality traits after merging genome-wide SNP association data with the copy number variation map [21]. To our knowledge, only one study has initiated CNV discovery in pigs that were selected from the two ends of the fat/lean estimated breeding value spectrum, in an attempt to identify candidate CNVs associated with fatness [23].

The goal of this study is to investigate the feasibility of identifying candidate CNVs related to fertility in a selected population of high and low fertility boars. Gene content and reproduction QTLs that are mapped to the positions of identified CNVs were analyzed.

## Results

### CNV analysis

Prior to CNV analysis several quality control steps were carried out. We have not identified any samples with outlier noise in the log R ratio values. We also checked another type of noise, the so called ’genomic waves’, that are variations of the signal intensity related to the genomic position of the probe, thus the composition of the DNA [24], and found no outlier wavy sample. The animals were selected from a large set of samples, which were not all genotyped at the same time, thus we performed principal component analysis to investigate potential batch effects. The PCA identified clear stratification of the data based on the date of array procedure. The effect of the fertility status and breed of animals were also PCA tested and no clustering was observed with any of these two parameters.

We chose to apply the two available segmentation options in the SVS software to explore putative CNVs. This algorithm - although widely used in human studies [25] - has not yet been applied to any data set generated on the Porcine SNP60k platform.

**Figure 1:**
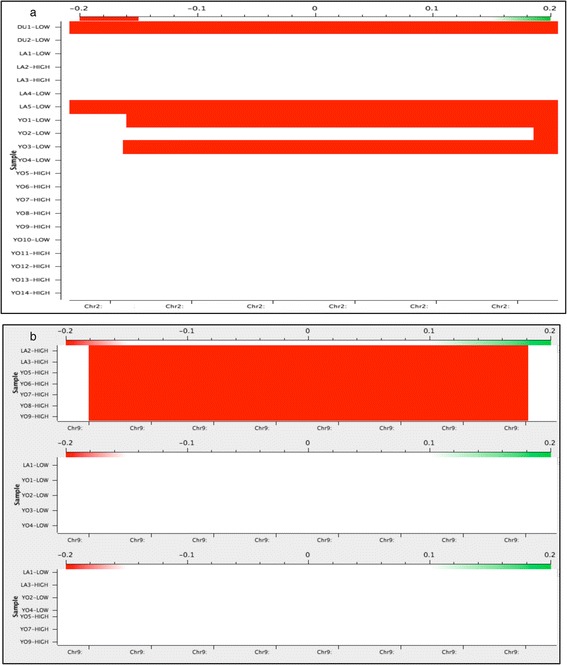
**Examples for the two CNV identification options in SVS software. a)** 21 individual animals genomes were subjected to the Univariate CNAM segmentation option. A red bar represents a segment with significantly lower log R ratio, as compared to its surrounding regions, thus identified as genomic loss. Slightly different length CNVs were identified in different low fertility samples, thus the overlapping region could be merged to a low fertility specific CNVR. **b)** The Multivariate CNAM method segments a group of samples together and segments are called only if present in all samples. In order to identify CNVs specific for either the high fertility or the low fertility group, the samples were segmented together, as well as grouped according to the fertility status. Only those CNVs were accepted that were present in only one group, but neihter in the other phenotypic group or in all samples together. Here the red bar identifies a CNVR specific for the high fertility group.

The first segmentation method (Univariate CNAM) searches individual genomes. Segments with significantly different log R ratio from its neighbors are identified as CNVs, which were then sorted according to the fertility status. Figure 1a shows a region of the genome where four CNVs of slightly different lengths were identified in four low fertility animals and marked by red bars representing losses. The overlapping region could then be merged to a low fertility specific CNVR. This method identified 48 CNVs in individual genomes, which were then compared to their fertility status and merged into 24 overlapping CNVRs. Among these, 10 were specific to the high fertility and 12 to the low fertility group, while two CNVR - although present in both groups - showed the opposite copy number status (loss in low fertility and gain in high fertility, or vice versa).

The second multivariate CNAM segmentation does not scan individual genomes, but rather checks if the segment cut-point is present in all samples for successful CNVR calling. We have used this approach to identify CNVRs specific for each fertility status by grouping the samples into low fertility or high fertility group and a 3rd control group contained all samples. As it is represented in Figure 1b, an acceptable high fertility group specific CNVR would be identified in all members of the given group, but should not be present in the low fertility group, neither in the control group. As the PCA identified samples clustering according to the date of genotyping, those two clusters were separated before multivariate CNAM to provide the maximal homogenous set of samples without confounding batch effects. We have discovered 11 CNVRs that fulfilled these criteria of multivariate CNAM, among those, four were specific to the high fertility group and seven to the low fertility group.

**Figure 2:**
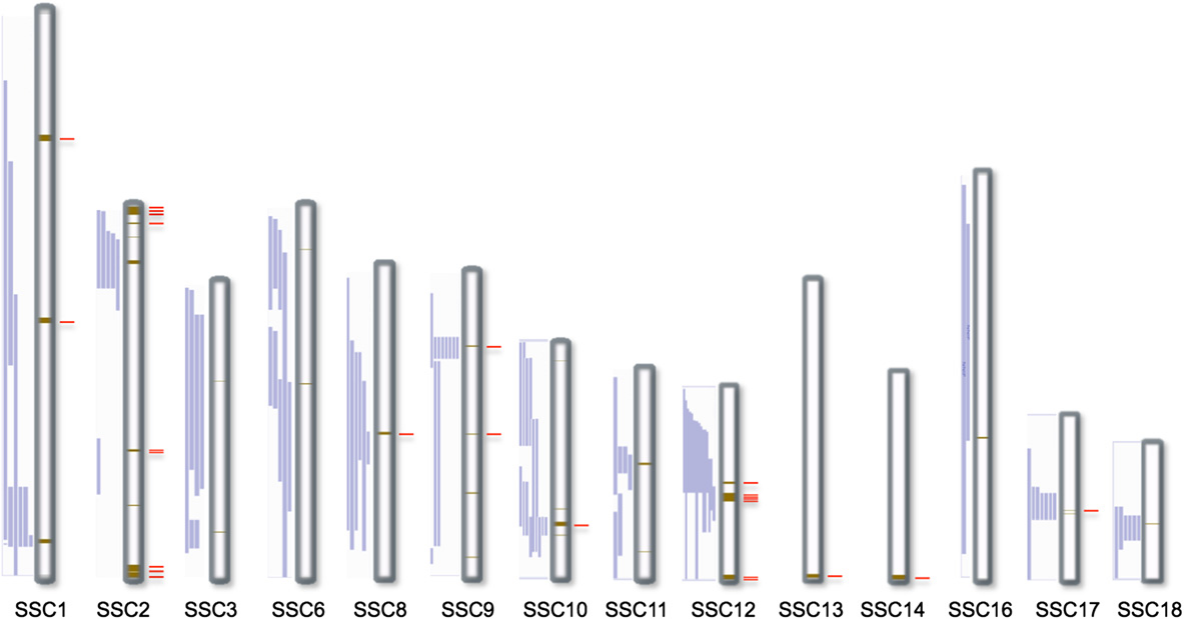
**Location of the detected CNVRs on the porcine chromosome ideograms.** The size of each ideogram is proportional to that of the chromosomes. The sex chromosomes were excluded from the analysis and no CNVR was detected on chromosome 4, 5, 7 and 15. The brown bars in the middle of each chromosome represent the positions of CNVRs. The purple columns in the left are the positions of QTLs and RefSeq genes are marked by red bars on the right side of the ideograms.

All together the two different strategies of CNAM resulted in the identification of 35 CNVRs. Fourteen CNVR were specific to the high fertility and 19 to low fertility boars (Additional file 1: Table S1). Only 14 of the 18 autosomes harbour CNVRs, as none was identified on chromosomes 4, 5, 7 and 15 (Figure 2). The name of each region, such as CNVR18L, is composed of ’CNVR’ followed by a number and ‘L’ for being specific to the low fertility group or ‘H’ in case of the high fertility group.

Chromosome 2 had the highest number of CNVRs (8) and the largest region involved in them (~12 Mb), while 5 other chromosomes had only 1–1 CNVR (Additional file 1: Table S2). We observed an excess of copy losses (28) and five gains and two regions where both gains and losses were found (Additional file 1: Table S3). The total length of CNVRs is approximately 36.5 Mb, which is distributed in a ratio of 4:1.5:1 among losses, gains and gain/loss regions.

**Figure 3:**
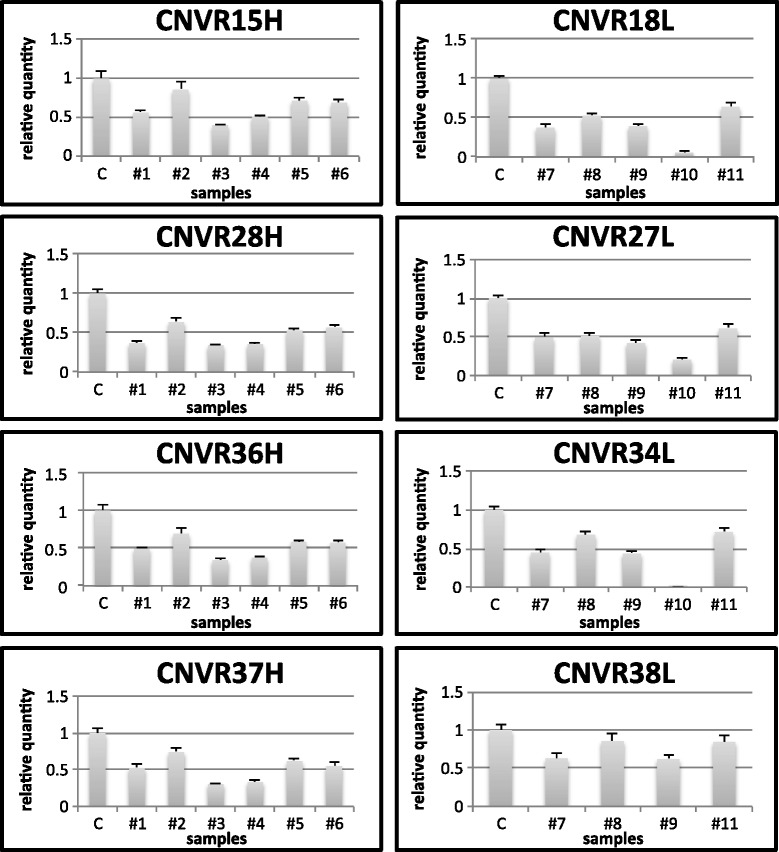
**Results of validation experiments for 8 CNVRs by qPCR. Relative quantity of target amplicons were calculated against the control sample (C) after normalization to the beta-actin locus.**

Quantitative real-time PCR (qPCR) was used to validate the identification of CNVRs. The results for all eight tested loci were in agreement with our predictions (Figure 3). Every animal from the group showed the predicted CNVR status in five CNVRs (CNVR18L, CNVR27L, CNVR28H, CNVR34L, CNVR36H), while five out of six samples tested positive at two loci (CNVR15H, CNVR37H) and two out of four samples were confirmed at CNVR38L.

Another aspect of validating the predicted CNVRs was to investigate an independent set of high and low fertile animals whether the same regions could be identified. In fact, 26 CNVRs were present. Seven of them have maintained significant association with the fertility status (CNVR5L, 7L, 10L, 18L, 45L, 50L, 62L). Interestingly, we found two regions (CNVR7L, 45L) where samples with both gain and loss status were present, although the original predictor set of animals represented only one of them.

### CNVRs overlapping reproduction QTL regions

The genomic positions of the 35 identified CNVRs were used to search for reproduction QTLs mapped to the same positions in the Animal QTLdb including the endocrine, litter size, reproductive organ and reproductive trait categories. The majority of CNVRs (30) overlapped with 137 QTLs from 16 traits and only five CNVRs are situated in regions of the porcine genome that have no reproduction QTL mapped (Figure 2, Additional file 1: Table S4). The chi-square test with Yates correction (p < 0.05) showed significant enrichment of reproduction QTLs among all QTL categories within the boundaries of the identified CNVRs.

The most abundant QTLs were the “teat number:TNUM” and “age at puberty:AGEP”. Thirty-eight TNUM and 26 AGEP were mapped to regions where CNVRs were detected. Twelve traits had QTLs mapped to chromosome regions where either low or high fertility group specific CNVRs were found, however the following 4 QTLs were found to be specific for only one of the fertility groups. A QTL for ‘plasma FSH concentration’ (QTL #646, [26]) was found to overlap with CNVR43H and CNVR44H from high fertility group animals. Two low fertility group specific CNVRs (CNVR13L and CNVR39L) overlapped with two different QTLs for ‘gestation length’ (#21837, [27]; and #452, [28]). One QTL for ‘testicular weight’ (#6527, [29]) harboured CNVR5L, a low fertility group CNVR. And at last, a ‘uterine capacity’ QTL (#523, [30]) lie together with the low fertility group specific CNVR34L.

### Functional annotation of CNVR gene content

Sequences - with RefSeq IDs - mapped to positions of CNVRs were retrieved from the UCSC Table browser. The identified 35 CNVRs encompassed 50 genes (Additional file 1: Table S1, S5). The majority of these were specific to the low fertility group (40), seven to the high fertility group, while three were found in regions that were present in both groups. Most of the genes, 27 and 10 respectively, were found on chromosomes 2 and 12, not surprisingly these two are covered with the longest, approximately half of the total size of CNVRs. CNVRs identified on chromosomes 3, 6, 11, 13 contain no genes.

A functional analysis of several databases revealed that the genes found in CNVRs from the low fertility group have been significantly enriched in members of the innate immune system, Toll-like receptor and RIG-I-like receptor signaling and fatty acid oxidation pathways (Table 1). The seven genes from the high fertility group CNVRs and the ones present in both groups do not specify any pathways with significant enrichment p-value.

**Table 1:** **Functional enrichment analysis of genes encompassing the identified CNVRs**

**Database**	**PathwayName**	**ID**	Statistics*	**Adjusted p-value**
KEGG	Toll-like receptor signaling pathway	04620	C = 102;O = 3;E = 0.10;R = 28.83;	0.0014
			rawP = 0.0002	
	Fatty acid metabolism	00071	C = 43;O = 2;E = 0.04;R = 45.59;	0.0032
			rawP = 0.0009	
	RIG-I-like receptor signaling pathway	04622	C = 71;O = 2;E = 0.07;R = 27.61;	0.0036
			rawP = 0.0024	
WikiPathways	Fatty Acid Beta Oxidation	WP143	C = 73;O = 3;E = 0.07;R = 40.28;	0.0005
			rawP = 5.86e-05	
	Toll-like receptor signaling pathway	WP75	C = 116;O = 3;E = 0.12;R = 25.35;	0.0008
			rawP = 0.0002	
	Regulation of toll-like receptor signaling pathway	WP1449	C = 154;O = 3;E = 0.16;R = 19.09;	0.0013
			rawP = 0.0005	
Pathway Commons	Immune System	522	C = 532;O = 6;E = 0.54;R = 11.05;	0.0012
			rawP = 1.62e-05	
	Innate Immune System	1094	C = 190;O = 4;E = 0.19;R = 20.63;	0.0012
			rawP = 4.32e-05	
	RIG-I/MDA5 mediated induction of IFN-alpha/beta pathways	1115	C = 67;O = 3;E = 0.07;R = 43.89;	0.0012
			rawP = 4.53e-05	
	Interferon Signaling	1123	C = 98;O = 3;E = 0.10;R = 30.00;	0.0013
			rawP = 0.0001	
	Toll Receptor Cascades	1095	C = 90;O = 3;E = 0.09;R = 32.67;	0.0013
			rawP = 0.0001	
	Interferon alpha/beta signaling	1122	C = 77;O = 3;E = 0.08;R = 38.19;	0.0013
			rawP = 6.87e-05	
	Toll Like Receptor 9 (TLR9) Cascade	1084	C = 65;O = 2;E = 0.07;R = 30.16;	0.0089
			rawP = 0.0020	
	Toll Like Receptor 2 (TLR2) Cascade	1136	C = 65;O = 2;E = 0.07;R = 30.16;	0.0089
			rawP = 0.0020	
	TRIF mediated TLR3 signaling	1074	C = 56;O = 2;E = 0.06;R = 35.01;	0.0089
			rawP = 0.0015	

*where C = number of reference genes in the category, O = observed number of genes in the gene set from the category, E = expected number in the category, R = Ratio of enrichment, rawP = p value from hypergeometric test, adjusted p-value = p value adjusted by the multiple test adjustment.

Five micro RNAs (miRNAs) were also found to position within CNVRs: miR-21, miR-142, miR-143, miR-145, miR-202 (Table 2). The latter was detected in both high and low fertility groups with opposite copy number status (deletion and gain, respectively), while the other four were only found in the low fertility group CNVRs, as deletions.

**Table 2:** **Summary of micro RNAs found within CNVRs**

**Name**	**Transcript ID**	**Chromosome**	**CNVR ID**	**Fertility**	**CNV status**
miR-21	NR_038508	12	CNVR50L	Low	DEL
miR-142	NR_038555	12	CNVR50L	Low	DEL
miR-143	NR_038529	2	CNVR16L	Low	GAIN
miR-145	NR_038484	2	CNVR16L	Low	GAIN
miR-202	NR_035399	14	CNVR59HL	High & Low	GAIN/DEL

## Discussion

In this study we applied the extreme groups/selective genotyping approach [31] for identifying copy number variations in high and low fertility breeding boars. These two groups of animals representing approximately 10% of both the upper and lower ends of the distribution from a large population of boars had mean DBE values of −2.7 and 2.8. One represents outstanding high fertility, while the others having high negative DBE values are considered low fertility. Animals from these two diverse phenotypes were compared by several approaches in order to prove the feasibility of our CNV analysis and to identify putative markers of fertility.

It should be noted that using a small subset of animals from the extreme ends of the phenotypic distribution not only reduce the cost of genotyping, but could retain the power of analysis as proven by simulation [31] and numerous QTL mapping studies [32]. Recently, it was also applied for CNV discovery as well, based on animals sampled from the distribution of fatness EBV [23].

We have identified 35 CNVRs covering 36.5 Mb or ~1.3% of the 2800 Mb porcine genome. The size range distribution of CNVRs is similar to that of other publications using the same SNP60k chip. There are numerous software tools available, such as PennCNV, cnvPartition, QuantiSNP, GADA to name a few which employ very different algorithms for the identification of CNVs from SNP array data [33-35]. A comparative analysis of several of these have found highly variable CNV calls due to this inherent difference [36]. Previous studies into porcine CNVs from SNP array data have chosen from these four softwares with slight preference to PennCNV [19-21,23]. However, we opted for the SNP and Variation Suite (SVS, GoldenHelix Inc.), mainly because the extensive tools available for quality assurance and the unique multivariate segmentation option providing the detection of associated regions across samples. Our motivation was to combine the advantages of using the extreme groups for the DBE phenotype and the increased analytical power of marker-level CNV test in detecting smaller common CNVs, as the latter was tested by Breheny et al. [37].

In fact, among the identified 35 CNVRs, 14 were specific to the high fertility animals, while 19 CNVRs were specific to the low fertility group, thus worth investigating their putative roles in fertility.

The quality of CNVR analysis was assessed by qPCR validation of four CNVRs specific for the low fertility and four for the high fertility group. All of the qPCR assays confirmed the CNV calls and 90% of the tested animals gave results in agreement with the prediction, that represents among the highest validation rate published to date in pigs [20]. We have also validated the identification of CNVRs using an additional independent set of high and low fertile boars. Our further analysis steps involved the comparison of CNVRs to already mapped reproduction QTLs, then the functional characterization of transcript content. Visual representation of these two comparisons is given in Figure 2, where the chromosomal positions of discovered CNVRs are aligned with QTLs and genes.

The identified CNVRs overlap with 137 QTLs of various reproductive traits (Additional file 1: Table S4.). QTLs generally represent the first estimation of the link between the genetic component of an important phenotypic variation and a smaller or larger segment of the genome [38]. These QTLs were identified and mapped with statistical significance by using various methods from microsatellite markers to whole genome scan on very different populations. The experiments led to the mapping of these 137 reproductive QTLs were published in more than a hundred studies, that could not be cited in this article, but could be accessed from the PigQTLdb [39]. Additional file 1: Table S1. contains all corresponding CNVR IDs and QTL IDs. The described CNVRs fall into the 7 kb to 1.6 Mb size range, that is in many cases much smaller than the current QTL region, thus could facilitate narrowing down the real functional locus and help the identification of the causative gene. It should also be mentioned that CNVRs described here were not tested for statistical association with QTLs, simply the overlapping genomic positions of the latter was used as one indicator of the potential function. Nonethless we found that reproduction QTLs were over-represented within CNVR boundaries.

As the porcine genome sequence and annotation are available in public databases [40], we attempted to characterize the functional content of CNVRs. One of the common result of pathway analysis using the various databases was the significant enrichment of elements of the innate immune system in low fertility samples (Table 1). A well-known connection exists between infections of either the female or male reproductive tract and impaired fertility. The innate immune system exhibits the non-specific response against pathogens, as the first-line of defense and then helps to activate the adaptive immune system. It is comprised of specific cell types, pattern recognition receptors and antimicrobial peptides, etc. Among these, we have identified CNVRs containing various components of the Toll-like receptor (TLR) signaling and RIG-I/MDA5 mediated induction of interferon signaling pathways. TLRs are transmembrane proteins that recognize pathogen associated molecular patterns. TLR2 binds those of microbes, while TLR3 is involved in cytoplasmic binding of viral nucleic acids [41], as well as RIG-I and MDA5 receptors [42]. These proteins are all localized throughout the male and female reproductive tract in humans and domestic species [41,43]. The balance of TLR response is required for physiological function of the reproductive organs - in order to protect against infections, and disturbed response has documented adverse effects on endometritis, ovulation, pregnancy outcome and sperm production [44,45].

Another significant pathway among the genes localized within CNVRs was the fatty acid metabolism. The presence and balanced metabolism of fatty acids are connected to a plethora of cellular functions, including the mitochondrial energy production, oxidative stress, cytoplasmic and membrane functions. These biological processes all affect fertility through the development of germ cells and their ability for successful fertilization. Fatty acids are metabolized in the mitochondria to produce acetyl-coA that enters the citric acid cycle and thus result in ATP. Motility of spermatozoa requires substantial energy resources [46] but the ATP level of the matured oocyte is also essential to provide energy for the developing embryo [47]. The cellular availability of different types of fatty acids contribute to the fluidity of plasma membrane, that is essential for cell fusion events, such as fertilization [48], but is also key to protect the cellular integrity from oxydative damage [49]. Three different CNVRs contain the following three members of this metabolic pathway. PNPLA2 codes an enzyme in the initial steps of lipid metabolism by catalyzing the hydrolysis of triglycerids and its impaired function was shown to result decreased plasma fatty acid levels [50]. Similarly, the product of CPT1A gene is key to the mitochondrial transport of long-chained fatty acids [51], while ECHS1 is the hydrolase in the second step of the beta oxidation, thus their functional imbalance affects the rate of fatty acid metabolism [52].

MicroRNAs (miRNAs) are key players in gene expression regulatory networks, as such, they might be strong candidates for disease-causing non-coding sequences. The variable dosage of miRNA genes due to their involvement in CNVs is affecting their expression profile and regulatory role [53]. Wu et al. [54] suggested an evolutionary mechanism that could correct for this by increasing the diversity of acting miRNAs on their targets and/or adjusting the copy numbers of their major target genes according to the CNV of the miRNA. The CNVRs found here harbor five miRNA genes (miR-21, miR-142, miR-143, miR-145, miR-202), the first four of them specific for the low fertility animals. Interestingly, none of their predicted target genes are situated within the boundaries of the 35 CNVRs described in this study. This would theoretically suggest the impaired function of these miRNAs and their putative role in the phenotype, although laboratory validation of their expression level is necessary to prove this. It is also interesting that these are among the most abundant miRNAs expressed in the male and female reproductive tissues [55,56]. miR-21 is present in testicular germ cells [57] and linked to the maintenance of spermatogenic stem cell population [58]. Furthermore, it is also localized in granulosa cells of pre-ovulatory follicles and plays a role in the follicular-luteal transmission, proven by its increasing level of expression [59]. Similarly, miR-142 shows variable expression levels between follicular and luteal phases [55]. miR-143, miR-145 are found to co-express and function in the regulation of cell proliferation [60], smooth muscle [61] and adipocyte development [62]. Some studies found these to be preferentially expressed in the male gonads [63] and epididymis [64] while others reported abundant expression in the ovary [56] and functions related to endometriosis [60].

miR-202 was identified as copy number gain in low fertility and deletion in the high fertility group, which would imply a negative role in fertility regulation. This is in agreement with the observations of its marked upregulation in various testicular hystopathologic conditions [65] and also in premature ovarian failure patients [66].

Similar to miR-202, we have found one gene with gain/deletion copy number status in CNVR1HL. However, it is a deletion in the low fertility and a gain in the high fertility group. Although this status distribution would make it an optimal marker for fertility, it was only found in 1 animal from each group. Furthemore one gene, the Gluthatione S-transferase mu2 (LOC780435, NM_001078684), is mapped to this region of chromosome 1. The superfamily of these metabolic enzymes functions as important players in protecting the cells from oxidative damage and endogenous toxicity [67]. Interestingly, in humans it lies in a hypervariable region, where structural rearrangements and deletions are frequent. The resulting variability in gene copy number, structure and enzyme activity thought to contribute to the individual’s stress response and strong association has been found with sperm production and male infertility [68].

## Conclusions

We have demonstrated that our analysis pipeline could identify putative CNV markers of fertility, especially in case of subfertile boars. Their relevance was demonstrated by analyzing the nature of co-localized reproductive QTLs and genes.

## Methods

### Animals and array genotyping

**Figure 4:**
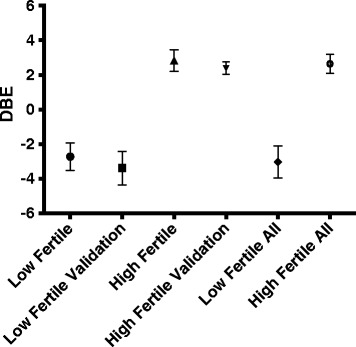
**Descriptive statistics (mean ± SD) of the Direct Boar Effects (DBE) in the high and low fertile animals, the validating groups and all together.**

The Canadian Centre for Swine Improvement Inc. [69], as a non-profit organization, collects and manages pedigree, breeding and performance information, as well as SNP genotypes of breeding animals from numerous major breeders across Canada to calculate and provide estimated breeding values for various traits. This integrated database was screened to identify boars with exceptional high and low fertility. The fertility indicator was defined as the calculated Direct Boar Effect on litter size (DBE) that can be obtained as a by-product of the national genetic evaluation for litter size (BLUP). DBEs are more accurate than only considering litter size averages in mates, since the estimated boar effects are then corrected for all identified environmental effects and breeding values of their mates [70]. The DBE value precisely shows how many more or less piglets a given boar produces per litter on average, as compared to the overall average of the population. The database contained 16,959 Yorkshire, 14,188 Landrace and 7366 Duroc boars with calculated DBE values. From these more than 38,500 boars we catalogued animals from the most extreme 10% on both sides of the distribution, that would be equal to approximately ±2 more or less piglets than herd average. Among these high and low fertile boars we have selected the ones which had the Porcine SNP60k array genotypes available, generated at the genomics facility in DNA LandMarks Inc. (QC, Canada), as part of large genotyping projects. For the purpose of CNV prediction 11 high fertility boars having 2.83 ± 0.61 (mean ± SD) DBE value and 10 low fertile boars with −2.72 ± 0.79 DBE value were randomly selected. Moreover, eight high fertile (DBE 2.38 ± 0.36) and 9 low fertile boars (DBE −3.38 ± 0.97) were randomly chosen to be used for validation of the CNV predictions. Distribution of DBE values of the various groups are presented in Figure 4. All together, the selected 19 high fertile and 19 low fertile animals represent various breeds, such as Yorkshire (21), Landrace (14) and Duroc (3).

### CNV analysis

Each probe-pair on the Illumina SNP60k genotyping array (for alleles A and B) marks a specific location in the porcine genome and its signal intensity could be related to the amount of DNA at that locus. In order to estimate DNA copy numbers, the observed normalized probe signals in each samples were compared to an expected signal intensity calculated from the Illumina defined reference sample cluster, thus generating a log R ratio value (log_2_(R_observed_/R_expected_), as described by Peiffer et al., [71]). This procedure was done using the Illumina GenomeStudio software, before being transferred into the SNP and Variation Suite version 7.7.8 (SVS, GoldenHelix) for quality control and CNV analysis.

Noise in the log R ratio values, inherent from the sample preparation or genotyping procedure could cause faulty identification of CNVs or confounding associations. Thus, our quality assurance workflow consisted of several different steps to identify potentially low quality samples. The X and Y chromosomal data were excluded from the data. Initial quality controls for noisy samples were done by testing for outliers in the median derivative log ratio values. Genomic wave factors were detected using the correction algorithm developed by [24] Diskin et al., as implemented in SVS. Potential batch effects were tested by principal component analysis (PCA) of logR values. SNP marker locations were annotated on the latest genome build, Sscrofa 10.2/susScr3 (2011).

There are two different Copy Number Analysis Methods (CNAM) in SVS, implementing the same segmenting algorithm two different ways. The Univariate CNAM scans each sample separately and ideal for identifying larger segments in individual genomes, while the Multivariate CNAM segments all samples simultaneously, thus generally smaller but common CNVs could be identified. We have applied both segmentation methods on our dataset to predict CNVs with maximum pairwise segment p value being 0.005, the min number of markers/segment value either 1 or 3 and the segment means were filtered to be < −0.15 for losses or >0.1 for gains. Overlapping CNVs were then merged to a CNV region (CNVR). The high and low fertility groups were also separated for the Multivariate CNAM, thus facilitating to identify CNVs specific for only one phenotype. The validation set of high and low fertile animals were segmented with the Univariate and Multivariate methods using the same conditions. The two-sided Mann–Whitney U-test were used to detect significant (p < 0.05) differences between the high and low fertile groups.

### qPCR validation

Eight CNVRs among the ones present in the largest number of animals (four CNVRs specific for the high and four CNVRs for the low fertility group) were validated by quantitative real-time PCR (qPCR). The DNA samples of the animals - in which the CNVRs were predicted - were retrieved from the DNA collection at our industry partner. No experiments were carried out on animals, thus no ethical approval was required. Primers were designed using the Primer3 plug-in of Geneious software. The primers by Chen et al. [20] were used for the beta-actin control region. Primer sequences and product sizes are in Additional file 1: Table S6. qPCR was performed using a CFX96 Touch™ Real-Time PCR Detection System (Bio-Rad) under the following thermal profile: 98°C, 2 min; 45 × (98°C, 10 sec; 59°C, 10 sec) followed by the registration of a melting curve between 68°C to 95°C in 0.5°C/sec increments. The 10 μl reaction was composed of 1× SsoFast EvaGreen Supermix (Bio-Rad), 3 mM primers and 20 ng genomic DNA. Samples were run in triplicate. Primer efficiencies were calculated as the average of individual well efficiencies determined by linear regression of amplification curves using the LinRegPCR software [72]. The relative quantity of each locus was determined against to the control sample after normalization to the beta-actin signal using the formula described by Pfaffl et al. [73].

### Functional annotation of CNVRs

Genomic locations of QTLs and the ones involved in reproductive traits (reproduction QTLs) were downloaded from the Animal Genome Database [39]. Enrichment of the latter in CNVR regions were tested using the Chi-square test with Yates correction. The RefSeq gene list was downloaded from the UCSC Table browser [74]. The resulting list of annotated genes was further analyzed for functional enrichment in Gene Onthology (GO) terms using the various tools implemented in the WEB-based Gene SeT AnaLysis Toolkit (WebGestalt, [75]). The porcine gene names were converted to the corresponding human ones and the resulting list was contrasted against the human genome as reference set for the default statistical test (Benjamini-Hochberg, adjusted p-value <0.01).

### Availability of supporting data

The data set supporting the results of this article is available in the NCBI's Gene Expression Omnibus [76] repository [GEO Series accession number GSE66170, http://www.ncbi.nlm.nih.gov/geo/query/acc.cgi?acc=GSE66170].

## Additional file

Additional file 1: Table S1. Details of the identified CNVRs. Table S2. Numbers and length of CNVRs are represented by chromosome. Table S3. Summary of CNVRs according to the copy number states. The number and length of CNVRs identified as Gain, Deletion or Gain/Del are given. Tabel S4. Summary of the number of reproduction QTLs overlapping with CNVRs. Table S5. Summary of gene content (RefSeq genes) within CNVRs specific for high fertility, low fertility animals or present in both. Table S6. Primer squences used for the qPCR validation experiments.

## Abbreviations

BLUP: Best linear unbiased prediction
CNV: Copy number variation
CNVR: Copy number variable region
CNAM: Copy number analysis method in SVS
DBE: Direct boar effect
PCA: Principal component analysis
QTL: Quantitative trait locus
SNP: Single nucleotide polymorphism
SVS: SNP and variation suite software (GoldenHelix Inc.)
